# Defect-Contributed Nonlinear Absorption Properties and Optical Limiting Potential of NaBi(WO_4_)_1.6_(MoO_4_)_0.4_ Single Crystals

**DOI:** 10.1021/acsomega.6c01109

**Published:** 2026-04-07

**Authors:** Gulden Yildiz Senguler, Anıl Dogan, Giray Mecit, Nizami Gasanly, Mehmet Isik, Ahmet Karatay, Ayhan Elmali

**Affiliations:** † Department of Physics Engineering, Graduate School of Natural and Applied Sciences, 37504 Ankara University, 06110 Ankara, Türkiye; ‡ Basic Sciences, TED University, 06420 Ankara, Türkiye; § Department of Physics, Science and Art Faculty, Middle East Technical University, 06800 Ankara, Türkiye; ∥ Virtual International Scientific Research Centre, Baku State University, 1148 Baku, Azerbaijan; ⊥ Department of Biomedical Engineering, Faculty of Engineering and Architecture, İzmir Bakırçay University, 35665 İzmir, Türkiye; # Department of Engineering Physics, Faculty of Engineering, Ankara University, 06100 Ankara, Türkiye; ¶ Institute of Artificial Intelligence, Ankara University, 06100 Ankara, Türkiye

## Abstract

In this study, the nonlinear optical response and optical limiting performance of a NaBi­(WO_4_)_1.6_(MoO_4_)_0.4_ single crystal grown by the Czochralski technique were systematically examined. The optical band gap of the synthesized crystal was evaluated as 3.33 eV, while the Urbach energy associated with structural defects, lattice disorders, and thermal effects was estimated to be 0.21 eV. Photoluminescence measurements reveal a wide emission band spanning the visible region from 400 to 700 nm, with pronounced emission peaks in the 438–486 nm range, indicating dominant near-band-edge recombination processes and confirming the direct band gap nature of the material. Nonlinear absorption characteristics were investigated using open-aperture Z-scan experiments under 532 nm excitation (photon energy of 2.32 eV). The results indicate that two-photon absorption (TPA) is the primary nonlinear mechanism governing the optical response. To elucidate the role of defect-related states, the experimental data were analyzed using two distinct theoretical approaches: a conventional TPA-based model and an extended model incorporating one-photon absorption, TPA, and free-carrier absorption. The effective nonlinear absorption coefficients obtained from the extended model were consistently higher than those derived from the TPA-only model, demonstrating that defect-assisted absorption processes significantly enhance the overall nonlinear response. Additionally, the NaBi­(WO_4_)_1.6_(MoO_4_)_0.4_ single crystal exhibits a low optical limiting threshold of 1.22 mJ/cm^2^ at the minimum incident energy density, highlighting its strong attenuation capability under high-intensity illumination. These findings suggest that this crystal is a promising candidate for optical limiting applications in the visible spectral range.

## Graphical abstract

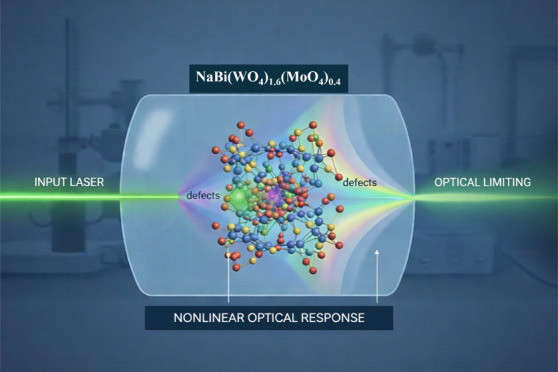

## 1 Introduction

Double tungstates and molybdates have attracted substantial interest owing to their versatile optoelectronic characteristics and broad applicability across photonic technologies.^1–4^ Among the members of this group, the materials NaBi­(MoO_4_)_2_ and NaBi­(WO_4_)_2_ are particularly interesting due to their magnetic, optical, and electrical properties.^5,6^ Numerous studies have demonstrated that phosphors obtained by incorporating rare-earth dopants into these hosts exhibit strong luminescence and can be integrated into devices such as LEDs, lasers, temperature sensors, gas detectors, and scintillators.^7,8^

Both compounds crystallize in a scheelite-type tetragonal structure (space group *I*4_1_/*a*), consisting of a three-dimensional framework of isolated [MO_4_]^2–^ (M = Mo, W) tetrahedra linked by Bi^3+^ and Na^+^ cations. Their lattice parameters have been reported as *a* = *b* = 0.527 nm and *c* = 1.158 nm for NaBi­(MoO_4_)_2_,^9^ with comparable values for NaBi­(WO_4_)_2._ ^10^ The presence of heavy Bi^3+^ ions combined with transition-metal-centered tetrahedra leads to strong polarizability, anisotropic bonding, and enhanced photoelastic and piezo-optic effects. Each Mo­(W) ion forms a tetrahedral unit with four oxygen atoms, giving rise to MoO_4_^2–^ (WO_4_^2–^) groups that play a central role in governing electronic transitions. The optical bandgaps of NaBi­(MoO_4_)_2_ and NaBi­(WO_4_)_2_ are typically reported around 2.90 and 3.50 eV, respectively.

Partially substituting Mo with W forms mixed-composition members, denoted as NaBi­(W_1–*x*_Mo_*x*_O_4_)_2_, enabling systematic tuning of structural and electronic features. Of particular interest is the composition *x* = 0.2 (NaBi­(WO_4_)_1.6_(MoO_4_)_0.4_), which combines the attributes of both end members. The most attractive feature of this solid-solution system is the ability to precisely modulate key optical parameters—most notably the bandgap, which can be engineered within the 2.90–3.50 eV range by adjusting the Mo/W ratio. Despite their potential, studies on the nonlinear optical and optoelectronic characteristics of NaBi­(W_1–*x*_Mo_*x*_O_4_)_2_ compounds remain relatively limited.^11^

Recent investigations have revealed that NaBi­(W_1–*x*_Mo_*x*_O_4_)_2_ crystals exhibit tunable nonlinear absorption and optical limiting behaviors, where defect-mediated electronic states play an enabling role in photonic device performance.^12^ NaBi­(MoO_4_)_2_ and NaBi­(WO_4_)_2_ belong to the scheelite-type molybdate–tungstate family, which has attracted considerable interest due to their structural stability and diverse optical and functional properties. The formation of mixed tungstate–molybdate compositions provides an effective route to tune the lattice structure and physical properties of these materials. Such compositional modifications may influence optical absorption behavior, lattice dynamics, and defect-related processes in these compounds.^13,14^ In crystalline materials, defect states can significantly influence optical and electronic properties by introducing localized energy levels within the band gap. These defect-induced midgap states may act as intermediate electronic levels that facilitate nonlinear optical interactions such as nonlinear absorption, optical limiting, and third-order nonlinear optical (χ^3^) processes.^15^ In mixed tungstate–molybdate systems, partial substitution between W^6+^ and Mo^6+^ ions can lead to local lattice distortions and the formation of intrinsic defects such as vacancies or substitutional disorder. These structural imperfections may slightly modify the electronic band structure and create additional defect-related states that contribute to defect-mediated optical responses. However, since the present compound crystallizes in the centrosymmetric *I*4_1_/*a* space group, second-order nonlinear optical effects such as second harmonic generation (χ^2^) are symmetry forbidden. Therefore, any defect-related enhancement is expected to influence mainly higher-order nonlinear optical responses rather than second-order processes. Therefore, NaBi­(W_1–*x*_Mo_*x*_O_4_)_2_ crystals are promising candidates for laser frequency doubling, ultrafast optical switching, and photonic data-storage applications.^16^ Rare-earth doping (e.g., Er^3+^, Yb^3+^, Ho^3+^) introduces additional degrees of freedom for tailoring emission pathways, enabling tunable luminescence suitable for LEDs, display technologies, and bioimaging materials.^9,17^ The scheelite structure supports extensive substitution without significant lattice disruption due to its high structural flexibility. Incorporation of smaller Mo^6+^ ions (0.41 Å) in place of W^6+^ (0.42 Å) results in slight unit-cell contraction and enhanced lattice distortion, which can modulate optical band edges and defect-related emission.^18^

NaBi­(W_1–*x*_Mo_*x*_O_4_)_2_ single crystals are commonly grown via Czochralski or top-seeded solution growth methods, yielding optically transparent, mechanically stable materials with low optical losses and uniaxial birefringence.^19,20^ Their wide transparency window, chemical robustness, and strong anisotropy make them well suited for next-generation photonic and optoelectronic devices, including UV–visible optical limiters, harmonic generators, and solid-state laser systems. Importantly, their lead-free composition and environmental benignity provide an eco-friendly alternative to conventional nonlinear optical crystals such as LiNbO_3_ and BaB_2_O_4_.^21,22^

In this work, the influence of defect-related energy states on the nonlinear absorption behavior of the NaBi­(WO_4_)_1.6_(MoO_4_)_0.4_ single crystal is systematically examined through the application of two distinct theoretical fitting approaches. Nonlinear optical measurements were carried out using the Z-scan technique under 532 nm excitation with nanosecond pulse durations. To complement these measurements and gain insight into the underlying carrier dynamics, ultrafast pump–probe spectroscopy was employed to probe charge-transfer processes on femtosecond to nanosecond time scales. Furthermore, the optical limiting capability of the crystal was evaluated to assess its potential for practical photonic protection applications.

## 2 Experimental Details

### 2.1 Synthesis of NaBi­(WO_4_)_1.6_(MoO_4_)_0.4_ Single Crystal

Single crystals of NaBi­(WO_4_)_1.6_(MoO_4_)_0.4_ were grown by the Czochralski technique. High-purity starting materials, namely Bi_2_O_3_ (99.9%), WO_3_ (99.9%), MoO_3_ (99.5%), and Na_2_CO_3_ (99.5%), supplied by Sigma-Aldrich, were used for crystal preparation. The raw materials were weighed according to the stoichiometric ratio 1Bi_2_O_3_ **:**3.2WO_3_ **:**0.8MoO_3_ **:**1Na_2_CO_3_, corresponding to the nominal composition of the crystal. The thoroughly ground powders were homogeneously mixed and subsequently pressed into pellets. These pellets were calcined at 800 °C for 20–24 h to ensure the formation of a single-phase precursor material. Crystal growth was carried out under controlled conditions with a pulling rate of 2 mm h^–1^ and a rotation speed of 15 rpm**.** The grown crystal ingot was cut into plate-like samples of surfaces perpendicular to the *c*-axis.

### 2.2 Characterizations

The crystal structure of the synthesized compound was examined using X-ray diffraction (XRD) analysis performed on a Rigaku Miniflex diffractometer equipped with CuKα radiation (λ = 0.154056 nm). The linear optical properties of the NaBi­(WO_4_)_1.6_(MoO_4_)_0.4_ single crystal were characterized using a UV–visible spectrophotometer (Shimadzu UV-1800). Ultrafast charge carrier dynamics were examined by femtosecond pump–probe spectroscopy. The experimental setup was based on a Ti:sapphire regenerative amplifier system (Spectra Physics, Spitfire Pro XP coupled with TOPAS), delivering laser pulses with a repetition rate of 1 kHz and an initial pulse width of 52 fs. An optical parametric amplifier was employed to tune the pump wavelength according to the crystal’s dominant linear absorption band. Within the pump–probe configuration, the effective pulse duration was measured to be approximately 120 fs through cross-correlation analysis. Transient absorption measurements were carried out over a temporal window extending from 0.1 ps to 3.2 ns, utilizing a white-light continuum probe generated in the Helios system (Spectra Physics). The acquired transient data was processed and analyzed using Surface Xplorer Software (Ultrafast Systems). The nonlinear optical response of the crystal was investigated via open-aperture Z-scan measurements. These experiments were conducted using a Q-switched Nd:YAG laser source (Quantel Brilliant) operating at a repetition rate of 10 Hz with a pulse duration of 4 ns. All nonlinear absorption measurements were performed at an excitation wavelength of 532 nm, with incident pulse energies varied systematically between 1 and 5 μJ. A linear motorized sample stage was used to move the sample along the focused laser beam axis. The beam waist (ω_0_) is 21 μm and Rayleigh range (*z*_R_) was found as 2.6 mm from the relation of *z*_R_ = πω_0_^2^/λ. The sample thickness is 2 mm, which is smaller than the Rayleigh range and therefore satisfies the thin-sample condition (*L* < *z*_R_), commonly approximated as *L* ≪ *z*_R_ for reliable Z-scan analysis. Thus, the thin-sample approximation required for Z-scan measurements is fulfilled, consistent with previous reports in the literature.^23^

## 3 Results and Discussion

### 3.1 Crystal Structure of NaBi­(WO_4_)_1.6_(MoO_4_)_0.4_ Single Crystal

The crystal structure and orientation characteristics of the NaBi­(WO_4_)_1.6_(MoO_4_)_0.4_ single crystal were examined by XRD analysis. The recorded diffraction pattern shown in Figure 1 exhibits two well-defined and intense reflections located at 2θ = 33.85 and 67.45°, which were indexed to the (200) and (400) crystallographic planes, respectively. The absence of additional diffraction peaks indicates a high crystalline quality and confirms the presence of a strong preferred orientation along the ⟨100⟩ direction. The dominance of the (200) and its higher-order harmonic (400) reflections suggests that the measured surface is highly oriented, exhibiting a single-crystal-like diffraction behavior rather than a typical polycrystalline response. Assuming a tetragonal crystal structure, the lattice parameter *a* (where *a = b*) was determined using the Bragg equation (*d*_200_ = λ/2sinθ) and the interplanar spacing of the (200) reflection. For the (200) plane, the interplanar distance was calculated as *d*_200_ = 2.6459 Å. In a tetragonal system, the lattice parameter is related to the (200) plane by *a* = 2*d*_(200)_, yielding a lattice constant of *a* = *b* = 5.292 Å. The calculated lattice parameter (*a* = 5.292 Å) lies between those reported for NaBi­(MoO_4_)_2_ (*a* = 5.267 Å)^24^ and NaBi­(WO_4_)_2_ (*a* = 5.30 Å),^19^ which confirms the formation of a solid solution between molybdate and tungstate tetrahedra and follows Vegard-type behavior. In comparison with the end-member compounds, the slight variation in the lattice parameter can be attributed to the partial substitution between W^6+^ and Mo^6+^ ions within the (WO_4_)/(MoO_4_) tetrahedral units. Due to the small difference in ionic radii between W^6+^ (0.60 Å) and Mo^6+^ (0.59 Å), the mixed composition may induce local lattice distortions and defect states, which slightly modify the lattice dimensions. The sharpness and intensity of the diffraction peaks further confirm the excellent crystallinity and structural integrity of the grown NaBi­(WO_4_)_1.6_(MoO_4_)_0.4_ single crystal.

**Figure 1:**
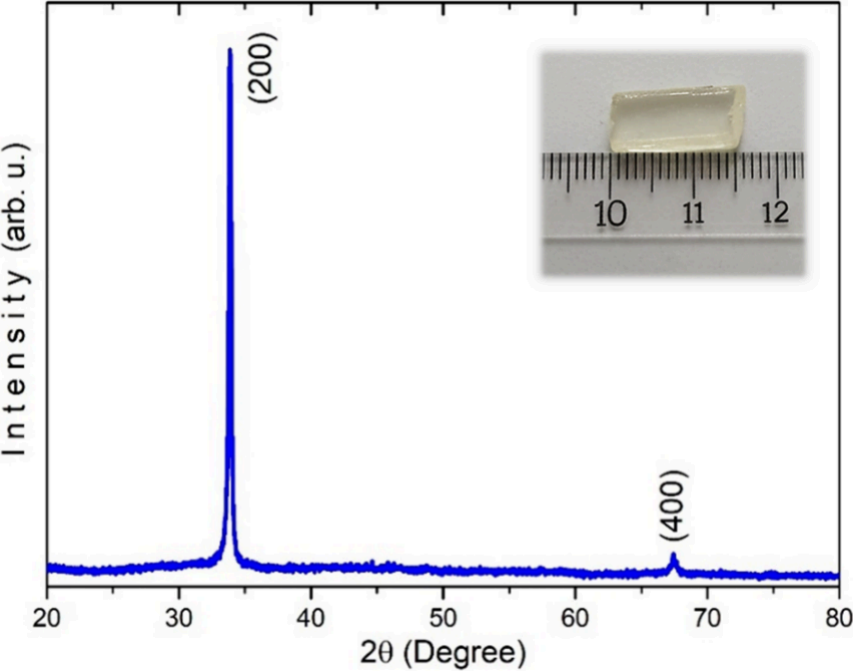
XRD pattern of NaBi­(WO_4_)_1.6_(MoO_4_)_0.4_ crystal.

### 3.2 Transmission and Photoluminescence Properties of NaBi­(WO_4_)_1.6_(MoO_4_)_0.4_ Single Crystal

The optical transmission characteristics of the NaBi­(WO_4_)_1.6_(MoO_4_)_0.4_ single crystal were investigated in the wavelength range of 300–800 nm. As seen from the transmission spectrum in Figure 2, the crystal exhibits a sharp absorption edge in the near-UV region, followed by a gradual increase in transparency toward the visible range. Such behavior is indicative of a wide-band gap oxide material with good optical quality and low defect-related absorption in the visible region. To accurately determine the optical band gap energy, the first derivative of the transmission spectrum with respect to wavelength (d*T*/dλ) was evaluated. The derivative spectrum reveals a pronounced peak centered at approximately 375 nm, which corresponds to the maximum rate of change in optical absorption and is commonly associated with the fundamental absorption edge. Using the relation *E*_g_ = *hc*/λ, the optical band gap of the NaBi­(WO_4_)_1.6_(MoO_4_)_0.4_ crystal was calculated to be approximately 3.31 eV. This value lies between the reported band gap energies of the end-member compounds, namely 2.90 eV for NaBi­(MoO_4_)_2_ and 3.50 eV for NaBi­(WO_4_)_2_, as available in the literature.^25,26^ The intermediate band gap obtained in this study is consistent with the partial substitution of MoO_4_^2–^ units by WO_4_^2–^ groups in the crystal lattice, leading to a gradual tuning of the electronic band structure. NaBi­(WO_4_)_1.6_(MoO_4_)_0.4_ crystal exhibits an optical response that bridges the gap between the molybdate- and tungstate-rich compositions, highlighting the effectiveness of compositional engineering in tailoring the optical properties of NaBi-based mixed oxide crystals.

**Figure 2:**
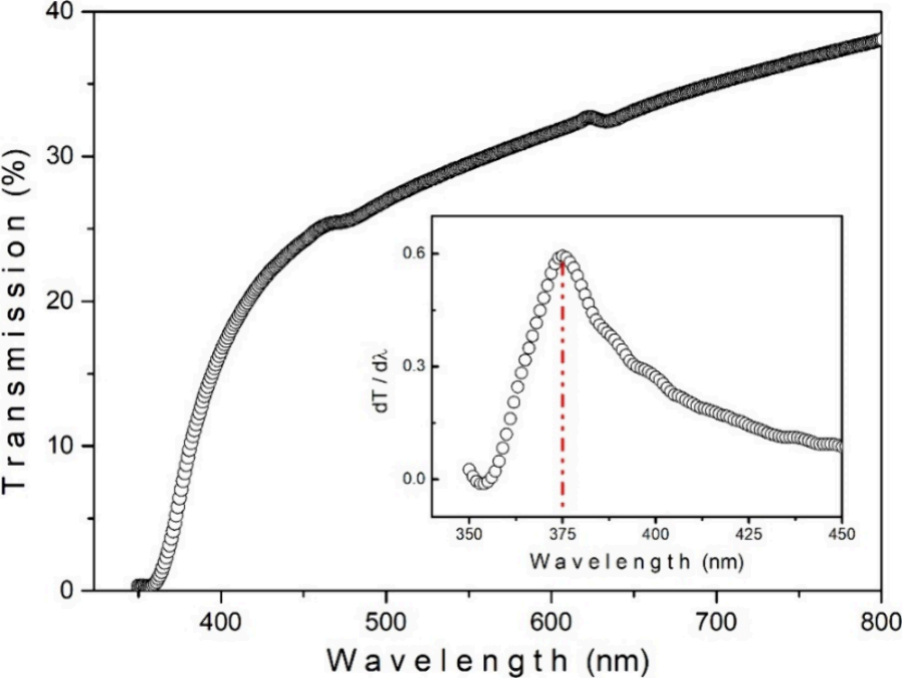
Optical transmission spectrum of the NaBi­(WO_4_)_1.6_(MoO_4_)_0.4_ single crystal. The inset shows the first derivative of the transmission spectrum (d*T*/dλ).

In order to further substantiate the optical band gap determination obtained from the derivative transmission method, the absorption coefficient (α) of the NaBi­(WO_4_)_1.6_(MoO_4_)_0.4_ single crystal was calculated from the measured transmission data and subsequently analyzed using the Tauc formalism. The absorption coefficient was evaluated assuming negligible reflection losses, which is a reasonable approximation for high-quality bulk single crystals with smooth surfaces. The optical band gap was determined by applying the Tauc relation,^27^

$$\left(\alpha hv{)}^{2}=A(hv-E_{g}\right)$$ 1

which corresponds to a direct allowed electronic transition. (α*hv*)^2^ as a function of photon energy (*hv*) exhibits a well-defined linear region near the absorption edge. Extrapolation of this linear portion to the energy axis shown in Figure 3 yields a band gap value of approximately 3.33 eV. This value is in excellent agreement with the band gap energy derived from the derivative transmission method (∼3.33 eV), confirming the reliability and consistency of the optical analysis. In addition, the optical disorder and band tailing effects in the crystal were evaluated using the Urbach rule, which describes the exponential absorption behavior near the band edge

$$\alpha =\alpha_{0}\exp(h\nu /E_{u})$$ 2

where *E*_U_ is the Urbach energy. The inset of Figure 3 shows the linear dependence of ln­(α) on photon energy in the low-energy absorption region. From the slope of the linear fit, the Urbach energy was determined to be 0.21 eV. The relatively low Urbach energy indicates a limited degree of structural disorder and a small density of localized states within the band gap, further supporting the high crystalline quality inferred from the XRD and transmission measurements. Overall, the close agreement between the band gap energies obtained from the derivative transmission method and the Tauc analysis, together with the low Urbach energy, demonstrates that the NaBi­(WO_4_)_1.6_(MoO_4_)_0.4_ single crystal possesses a well-defined electronic band structure with minimal defect-induced tail states.

**Figure 3:**
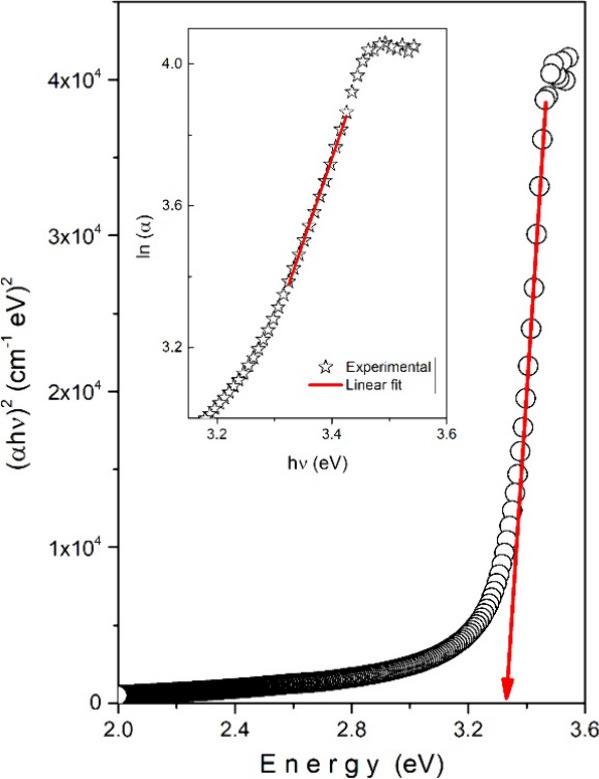
(α*hv*)^2^ vs *hv* plot for Tauc analysis. Inset indicates the Urbach analysis plot.

Figure 4a presents the room-temperature photoluminescence (PL) spectrum of the NaBi­(WO_4_)_1.6_(MoO_4_)_0.4_ single crystal was obtained under 400 nm wavelength excitation. The spectrum exhibits several distinct emission bands indicating that radiative recombination proceeds through multiple channels. When converted into photon energies, the observed emissions correspond to ∼2.75 eV (450.5 nm), 2.68 eV (463 nm), 2.55 eV (486 nm), and 2.33 eV (533.5 nm), all of which are below the optical band gap determined from absorption analyses (*E*_g_ ≈ 3.33 eV). Therefore, the observed PL features are predominantly attributed to sub-bandgap radiative transitions mediated by defect- or impurity-related states rather than purely band-to-band recombination. The set of emissions in the blue region (≈450–486 nm) may arise from recombination involving shallow donor/acceptor levels or excitonic-related processes assisted by localized states. In mixed tungstate–molybdate crystals, such sub-bandgap blue emissions have commonly been associated with intrinsic lattice imperfections and local structural distortions, including oxygen-related defects and cation-site perturbations.^28–30^ The broader green emission centered near 533.5 nm is characteristic of deeper defect-related transitions and can be linked to oxygen vacancies, localized states associated with Bi^3+^, and/or (W/Mo)­O_4_^–^ related defect complexes, which provide recombination pathways through deep levels.^28,30^ The coexistence of multiple emission peaks suggests the presence of multilevel recombination mechanisms, such as donor–acceptor pair (DAP) transitions and/or self-trapped exciton (STE)-assisted emission, which are frequently reported in complex oxide lattices where electron–phonon coupling and local disorder may stabilize localized excited states.

**Figure 4:**
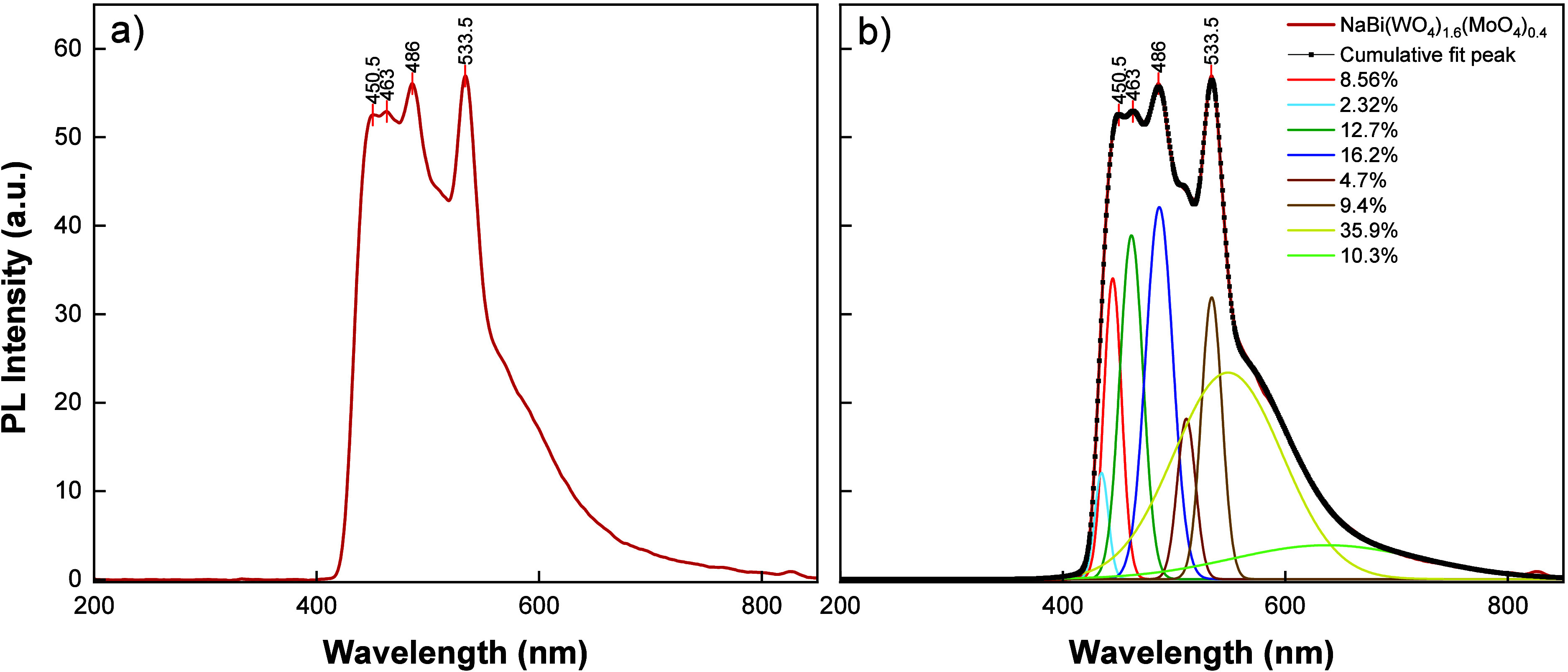
(a) Room-temperature PL spectrum of NaBi­(WO_4_)_1.6_(MoO_4_)_0.4_ single crystal (λ_exc_ = 400 nm), (b) Gaussian deconvolution of the PL spectrum, showing the individual emission components along with the overall fitted curve.

PL spectrum of Figure 4b presents a detailed analysis of the PL spectrum using Gaussian deconvolution. The black curve represents the experimentally obtained total PL spectrum, while the colored curves correspond to the individual emission bands resolved from this spectrum. The presence of multiple peaks indicates that the emission originates from several energy levels rather than a single mechanism. This analysis demonstrates that the optical emission of the material is strongly influenced by defect states, and that the observed PL behavior results from the superposition of multiple transition processes.

### 3.3 Ultrafast Transient Absorption Spectroscopy

Figure 5a presents the transient absorption spectra of the NaBi­(WO_4_)_1.6_(MoO_4_)_0.4_ single crystal recorded at different pump–probe delay times ranging from 102 fs to 3.14 ns. A pronounced excited-state absorption (ESA) signal is observed over the entire visible region (≈430–820 nm), indicating the generation of long-lived excited-state species following ultrafast photoexcitation. The magnitude of the absorption change is highest at early delay times (subpicosecond regime) and gradually decreases with increasing delay, reflecting relaxation and recombination processes of photoexcited carriers. The broadband nature of the ESA signal suggests the involvement of defect-related or charge-transfer states rather than narrow excitonic transitions. As the delay increases from the femtosecond to nanosecond time scale, the overall spectral shape remains largely preserved, while the amplitude decreases, indicating that the relaxation is governed primarily by population decay rather than spectral evolution.

**Figure 5:**
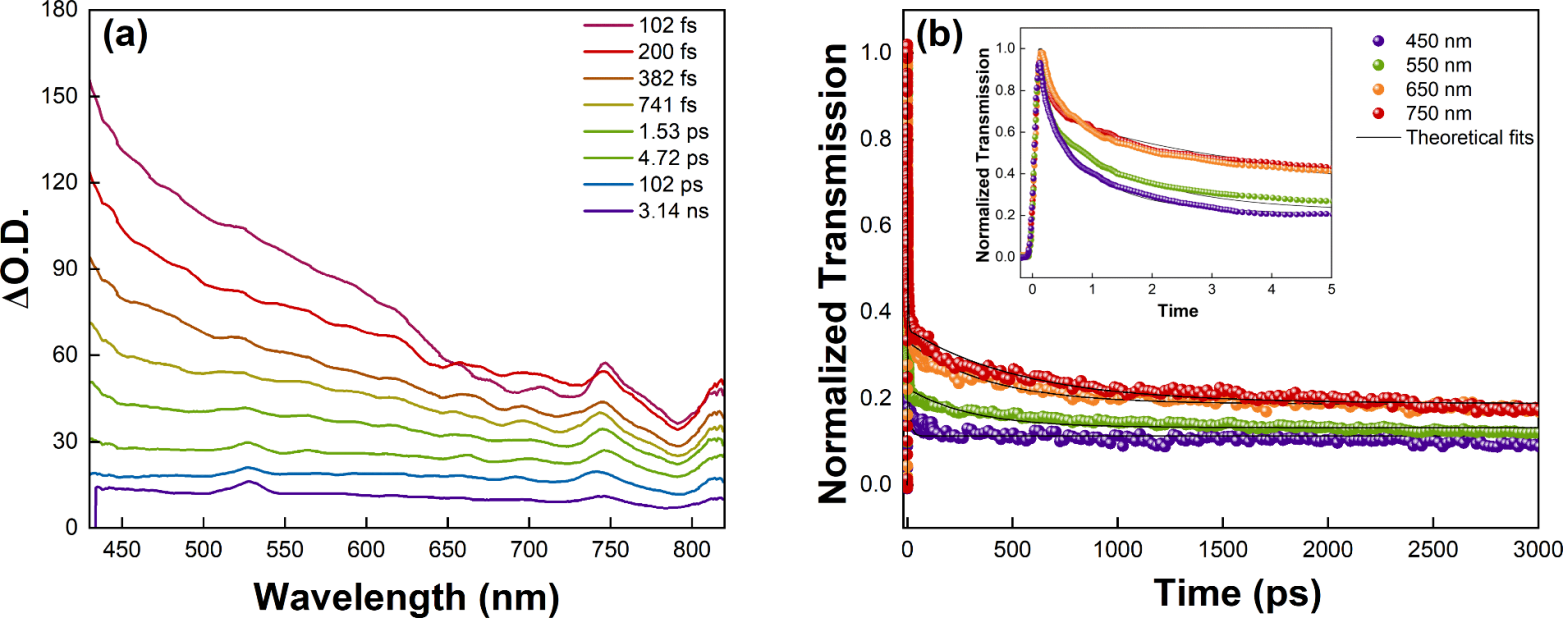
(a) Transient absorption spectrum of NaBi­(WO_4_)_1.6_(MoO_4_)_0.4_ single crystal at different time delays and (b) decay kinetics at 400 nm.

Figure 5b shows the normalized transient transmission dynamics measured at selected probe wavelengths (450, 550, 650, and 750 nm). All kinetic traces exhibit a rapid initial decay followed by a slower relaxation component, revealing multitime scale carrier dynamics. The ultrafast decay component in the subpicosecond to few-picosecond range can be attributed to carrier thermalization and trapping into defect or localized states. The subsequent slower decay, extending into the nanosecond regime, is associated with the recombination of trapped carriers or long-lived charge-transfer states. Notably, the decay becomes progressively slower at longer probe wavelengths, indicating wavelength-dependent relaxation dynamics. This behavior suggests that lower-energy probe photons are more sensitive to deeper trap states or defect-assisted transitions, which possess longer lifetimes. The good agreement between the experimental data and the theoretical fits confirms the validity of the adopted kinetic model and supports the presence of multiple relaxation channels.

### 3.4 Z-Scan Analysis of NaBi­(WO_4_)_1.6_(MoO_4_)_0.4_ Crystal

OA Z-scan measurements are conducted at 532 nm irradiation wavelength under several input densities to bring out the NA properties of NaBi­(WO_4_)_1.6_(MoO_4_)_0.4_ single crystal. OA Z-scan curves were adapted according to two different models. The results of the Z-scan measurements are given below. OA Z-scan measurements are conducted at 532 nm irradiation wavelength under several input densities to bring out the NA properties of NaBi­(WO_4_)_1.6_(MoO_4_)_0.4_ single crystal. The results of the Z-scan measurements are given in Figure 6.

**Figure 6:**
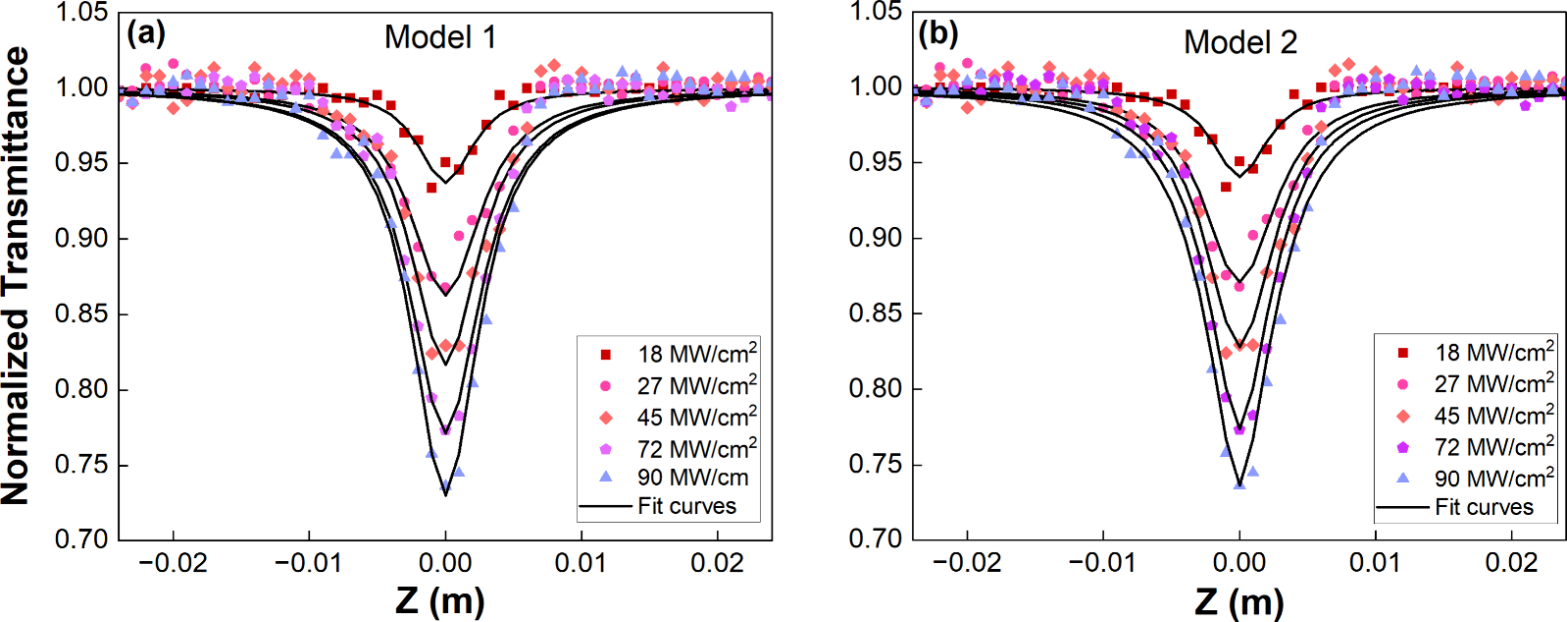
Theoretical fit curves of Z-scan data obtained from (a) model 1 and (b) model 2 with respect to input intensities.

**In model 1,** the optical nonlinearity can be defined in the following equations,^31^

$$\alpha (I)=\alpha_{0}+\beta I$$ 3

where α_0_ and β are linear absorption and TPA coefficients, respectively. The transmittance can be given by the eq 4,

$$T(z,S=1)=\frac{1}{\sqrt{\pi \rho_{0}(z,0)}}\int_{-\infty }^{\infty }\ln[1+\rho_{0}(z,0)e^{-\tau^{2}}]d\tau$$ 4

where ρ_0_(*z*,0) = β*I*_0_ *L*_eff_/(1 + *z* ^2^/*z*_0_^2^), *z* is the position of the material and *z* = 0 at he focus, *z*_0_ = *k*ω_0_^2^/2 is the Rayleigh range, ω_0_ is the beam waist at focus, *I*_0_ is the intensity of laser beam at the focus, and *L*_eff_ is the effective thickness of the material and given as *L*_eff_ = [1 – *e* ^–α_0_ *L*^]/α_0_, *L* is the thickness of the material.

**In model 2**, to indicate the effects of defect states on the NA, the Z-scan data was also fitted by using a theoretical model which includes the OPA, TPA, FCA, and their saturations given in following equations.^32^

$$\frac{dI}{dz^{\prime }}=-\frac{\alpha I}{1+I/I_{\mathrm{SAT}}}-\frac{\beta I^{2}}{1+I^{2}/I_{\mathrm{SAT}}^{2}}-\frac{\sigma_{0}\Delta NI}{1+I^{2}/I_{\mathrm{SAT}}^{2}}$$ 5

where *I* is the output intensity that comes from the sample to the detector, *z* ^′^ is the propagation distance of light inside the sample, *I*_SAT_ is the intensity threshold of saturation, β is the TPA coefficient, σ_0_ is the free-carrier absorption cross-section. Δ*N* is the photocarrier density that is a function of α and β, and it describes the light absorption of photocarriers in defect states via OPA, provided that the lifetime of the defect states is longer than the pulse duration. Δ*N* can be given as follows:

ΔN=(στ0ℏω0)I 6

where τ_0_ is the pulse duration and ω_0_ is the beam waist at the focus. Thus, eq 5 becomes the following equation.

$$\frac{dI}{dz^{\prime }}=-\frac{\alpha I}{1+I/I_{\mathrm{SAT}}}-\frac{\beta_{\mathrm{eff}}I^{2}}{1+I^{2}/I_{\mathrm{SAT}}^{2}}=-f(I)$$ 7

and

βeff=β+(σ0ατ0ℏω0) 8

where β_eff_ is a free parameter obtained from the fitting of the experimental data.

In order to reveal the contributions of defect states to nonlinear absorption (NA) through one-photon absorption (OPA) and ESA mechanisms, OA Z-scan data were fitted with model 1 and model 2 detailed above. Therefore, the NA coefficients (β) and (β_eff_) were obtained from the theoretical fits of the Z-scan data by using eqs 4 and 5, respectively. The results are listed in Table 1. Due to the lower energy of the incident light (2.32 eV) compared to the bandgap energy of the single crystal (3.33 eV), TPA is allowed.^33^ However, standard Z-scan measurements alone cannot reveal the exact nature of the TPA process, making intensity-dependent NA assessment necessary.

**Table 1:** Nonlinear Absorption Coefficients (β and β_eff_) and I_SAT_ Values with Excitation Parameters (λ, τ, and w_0_)

sample	λ_exc_ (nm)	τ_pulse_ (ns)	*w*_0_ (μm)	*I*_0_ (MW/cm^2^)	β (m/W) (model 1)	β_eff_ (m/W) (model 2)	*I*_SAT_ (W/m^2^) (model 2)	refs.
NaBi(WO_4_)_1.6_(MoO_4_)_0.4_	532	4	21	18	4.46 × 10^–10^	2.69 × 10^–9^	1.96 × 10^11^	This study
				27	8.92 × 10^–10^	2.81 × 10^–9^	2.14 × 10^11^	
				45	7.07 × 10^–10^	1.79 × 10^–9^	3.53 × 10^11^	
				72	6.02 × 10^–10^	1.17 × 10^–9^	5.71 × 10^11^	
				90	5.62 × 10^–10^	1.03 × 10^–9^	6.76 × 10^11^	
NaBi(MoWO_4_)_2_	532	4		66.16		1.24 × 10^–9^	1.80 × 10^11^	^12^
NaBi(Mo_0.5_W_0.5_O_4_)_2_						2.98 × 10^–9^	3.86 × 10^11^	
Bi_12_SiO_20_	532	4		36.10		3.98 × 10^–9^	4.46 × 10^11^	^36^
MoS_2_-24	532	9			2.3 × 10^–10^			^23^
rGO	532	CW		50	5.88 × 10^–5^			^37^
GO	532	CW		50	6.06 × 10^–5^			^38^
CdFe_2_O_4_-rGO (15 wt %)	532	CW		50	5.97× 10^–5^			

Between the two proposed approaches, model 2 provides a more accurate and physically meaningful representation of the crystal’s nonlinear optical behavior for several key reasons. Unlike model 1, which assumes a conventional and instantaneous TPA process, Model 2 explicitly accounts for the presence of midgap defect states that actively govern the material’s optical response. Model 2 successfully incorporates a sequential absorption mechanism. As illustrated in Figure 7, one photon is initially absorbed to populate real defect states via OPA, followed by the absorption of a second photon to reach higher excited states via ESA.^15,34,35^ Finally, the β_eff_ values obtained using model 2 are significantly higher than the β values derived from model 1 and demonstrate a clear intensity dependence. Specifically, NA decreased with increasing input densities because the defect states corresponding to the 2.32 eV energy region are filled by OPA (2.69 × 10^–9^ → 1.03 × 10^–9^ m/W). This confirms that sequential absorption mechanisms occurring through localized defect levels strongly reinforce the nonlinear optical response, making the extended theoretical framework of model 2 essential for an accurate description of the system.

**Figure 7:**
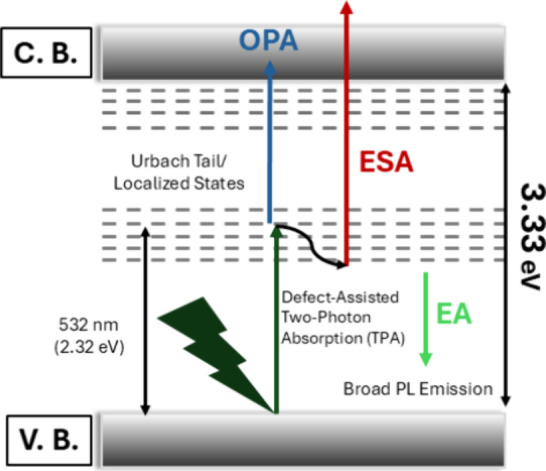
Energy-level diagram illustrating one-photon absorption (OPA), excited-state absorption (ESA), and emission/luminescence (EA) processes responsible for the nonlinear optical response and optical limiting behavior.

### 3.5 Optical Limiting Performance

The optical limiting quality of a material is determined by a threshold that corresponds to a fluence value where the normalized transmittance curve begins to decrease. Consequently, the threshold value must be as low as possible for an optical limiter of high quality. As illustrated in Figure 8, the normalized transmittance curves are shown as a function of distance-dependent fluence of the sample studied at a wavelength of 532 nm. The distance-dependent fluence relation is given by the following equation,^39^

$$I(z)=\frac{E}{\pi \omega^{2}(z)t}$$ 9

where *E* is the input energy per pulse and *t* is the pulse duration, *w*(*z*) is the beam waist as a function of distance and it can be determined by following the relation for Gaussian beam waist,

$$\omega^{2}(z)=\omega_{0}^{2}\left(1+\frac{z^{2}}{z_{R}^{2}}\right)$$ 10

and

$$z_{R}=\frac{\pi \omega_{0}^{2}}{\lambda }$$ 11

where *z*_R_ is the Rayleigh distance, λ is the wavelength of the incident light, and ω_0_ is the beam waist at the focal point. The optical limiting threshold of NaBi­(WO_4_)_1.6_(MoO_4_)_0.4_ was determined to be 1.22 mJ/cm^2^ at an incident intensity of 27 MW/cm^2^, indicating an efficient nonlinear response under visible laser excitation. A comparative analysis of optical limiting thresholds at 532 nm for NaBi­(WO_4_)_1.6_(MoO_4_)_0.4_ and previously reported single crystals is summarized in Table 2. The results clearly demonstrate that the NaBi­(WO_4_)_1.6_(MoO_4_)_0.4_ single crystal exhibits a comparatively low optical limiting threshold, suggesting enhanced optical limiting performance. This behavior may be attributed to the combined effects of nonlinear absorption processes and defect-related states, which enhance intensity-dependent attenuation of the incident beam. Consequently, NaBi­(WO_4_)_1.6_(MoO_4_)_0.4_ emerges as a promising candidate for optical limiting and laser protection applications in the visible spectral region.

**Figure 8:**
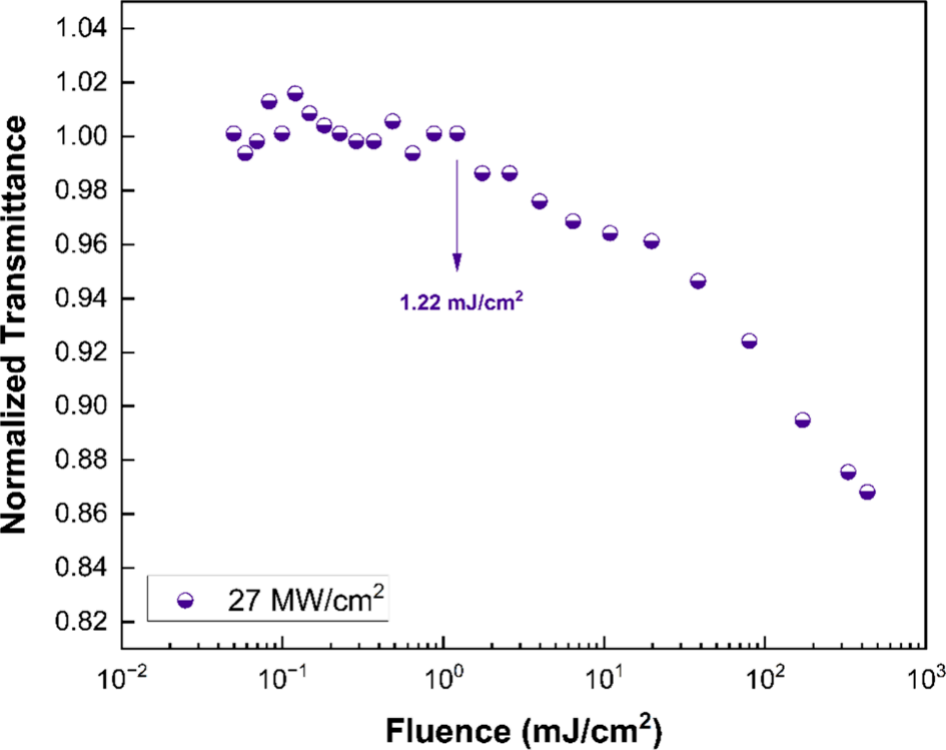
Optical limiting curve of NaBi­(WO_4_)_1.6_(MoO_4_)_0.4_ single crystal at 27 MW/cm^2^ intensity under excitations of 532 nm wavelength.

**Table 2:** Comparison of Optical Limiting Thresholds of NaBi­(WO_4_)_1.6_(MoO_4_)_0.4_ and Single Crystals in the Literature at 532 nm Wavelength

single crystals	optical limiting thresholds	references
NaBi(WO_4_)_1.6_(MoO_4_)_0.4_	1.22 mJ/cm^2^	Present work
NaBi(WO_4_)_2_	0.05 mJ/cm^2^	^12^
NaBi(Mo_0.5_W_0.5_O_4_)_2_	0.34 mJ/cm^2^	
NaBi(Mo_0.75_W_0.25_O_4_)_2_	0.44 mJ/cm^2^	
Bi_12_SiO_20_	0.34 mJ/cm^2^	^36^
Bi_12_TiO_20_	0.34 mJ/cm^2^	^40^
PbMoO_4_	4.91 mJ/cm^2^	^41^
PbMo_0.75_W_0.25_O_4_	3.45 mJ/cm^2^	^42^
MINP	1.79 × 10^12^ W/m^2^	^43^
MTPB	3.26 × 10^12^ W/m^2^	^44^
DMMA	1.3 kJ cm^–2^	^45^
2A5NPTCA	6.7 mW/cm^2^	^46^
GuZT	27.4 mW/cm^2^	^47^
DADLMA	3.34 × 10^–3^ W/cm^2^	^48^

## 4 Conclusions

In this work, the nonlinear absorption (NA) and optical limiting (OL) behaviors of NaBi­(WO_4_)_1.6_(MoO_4_)_0.4_ single crystal synthesized via the Czochralski growth method were systematically investigated. Owing to its band gap of 3.33 eV, the crystal exhibits pronounced two-photon absorption (TPA) under 532 nm laser excitation, where the photon energy (2.32 eV) is insufficient to induce direct one-photon transitions. A detailed analysis reveals that the inclusion of defect-related energy states significantly enhances effective NA coefficients. This enhancement is attributed to excited-state absorption (ESA) processes, where carriers initially promoted to defect levels through one-photon absorption further participate in ordered TPA mechanisms, leading to stronger absorption at elevated excitation intensities. Furthermore, the crystal demonstrates an intensity-dependent transition toward saturable absorption (SA), indicating that the observed nonlinear response arises not only from conventional TPA but also from the substantial contribution of defect-assisted ordered TPA processes. The coexistence of NA and SA behaviors highlights the complex carrier dynamics governing the nonlinear optical response of the NaBi­(WO_4_)_1.6_(MoO_4_)_0.4_ single crystal. The optical limiting performance in the visible spectral region confirms the practical applicability of this material for photonic protection systems. An optical limiting threshold of 1.22 mJ/cm^2^ was achieved at an incident intensity of 27.4 MW/cm^2^, demonstrating competitive performance relative to previously reported materials. Overall, the combined presence of strong NA for optical limiting and SA for intensity-dependent transmission modulation renders NaBi­(WO_4_)_1.6_(MoO_4_)_0.4_ versatile candidate for applications in visible-light optical limiting, optical switching, and advanced optoelectronic devices.
